# Experience of Violence and Socioeconomic Position in South Africa: A National Study

**DOI:** 10.1371/journal.pone.0001290

**Published:** 2007-12-12

**Authors:** Katherine Doolan, Rodney Ehrlich, Landon Myer

**Affiliations:** 1 School of Public Health and Family Medicine, Faculty of Health Sciences, University of Cape Town, Cape Town, South Africa; 2 Center for International Data Evaluation and Analysis, Women's Global Health Imperative, University of California at San Francisco, San Francisco, California, United States of America; 3 Department of Epidemiology, Mailman School of Public Health, Columbia University, New York, New York, United States of America; McGill University, Canada

## Abstract

**Background:**

Violence is a leading cause of morbidity and mortality in South Africa and needs to be researched from a public health perspective. Typically in violence research, socioeconomic position is used in the analysis to control for confounding. Social epidemiology approaches this variable as a primary determinant of interest and is used in this research to better understand the aetiology of violence in South Africa. We hypothesised that measures of socioeconomic position (employment, education and household wealth) would be inversely related to violence at the individual and household levels.

**Methodology/Principal Findings:**

Data came from the1998 South African Demographic and Health Survey (SADHS). Measures of socioeconomic position used were employment, education and household wealth. Eighty-eight people (0.2%) received treatment for a violent injury in the previous 30 days and 103 households (0.9%) experienced a violent death in the previous year. Risk factors for violence at the individual level included employment (41% of those who experienced violence were employed vs. 27% of those who did not, p = 0.02), and education (those who experienced violence had on average, one year more education than those who did not, p = 0.04). Belonging to a household in the wealthiest quintile was protective against violence (OR: 0.32; 95% CI: 0.12–0.89). In contrast, at the household level all three measures of socioeconomic position were protective against the experience of a violent death. The only association to persist in the multivariate analysis was that between the wealth of the household and violence at the individual level.

**Conclusions/Significance:**

Our hypothesis was supported if household wealth was used as the measure of socioeconomic position at the individual level. While more research is needed to inform the conflicting results observed between the individual and household levels, this analysis has begun to identify the disparities across the socioeconomic structure with respect to violent outcomes.

## Introduction

There is increasing recognition of violence as a preventable source of morbidity and mortality, particularly in developing country settings. Globally, the estimated mortality rate due to violence was 28/100,000 in 2000 [1]. In South Africa, where violence is the second leading cause of premature death, this rate is 73/100,000 [2], and homicide accounts for 56% of fatal injuries among individuals 15–34 years of age [3]. Clearly violence is a significant public health concern: beyond the direct effect of violent injury on health, both victims and witnesses of violence may experience emotional trauma and long-term psychological effects of violence [4].

There is a long history in sociology of viewing social and economic conditions as general determinants of violent behaviour [5]–[7], but only relatively recently have epidemiologists come to view socioeconomic factors as explanatory variables contributing to the causation of violence and injury, rather than as variables which simply confound other associations [8]. In a meta-analysis of 34 international aggregate data studies (primarily from developed countries), Hsieh and Pugh found that of the 41 correlation coefficients for poverty and various types of violent crime, 32 (or 78%) were of at least moderate strength (>0.25) [9]. However, others have stressed that the severity of the outcome (fatal or nonfatal injuries) and the specific measurement of socioeconomic position (education, employment, income, wealth) used in the analysis may have a substantial effect on the relationship between socioeconomic position and injury [10]. The complexity of the relationship between violence and social position requires that research on the aetiology of violence extend beyond the fields of criminology and law (the traditional academic centres of violence research) to include epidemiological perspectives. In particular, social epidemiology may offer the ability to better understand how different social and economic factors are involved in the aetiology of violence in different settings.

Despite the importance of violence as a public health problem in developing countries, and general interest in socioeconomic factors as determinants of violence, there have been few population-based studies investigating the aetiology of violence outside of Europe and North America. The majority of epidemiological research into violence in developing countries has focused on small geographic areas, or specific topics such as violence against women [11]. Given the burden of disease associated with violence in South Africa and other developing countries, there is a need for epidemiological research into the determinants of violence in these settings.

The South African Demographic and Health Survey (SADHS) offered an opportunity to explore some of these questions [12]. Specifically, it enabled us to test the association between different measures of socioeconomic position and violence, and do so at both individual and household levels. As outcomes we used the receipt of treatment for a violent injury at the individual level and a household member's death due to violence at the household level. We hypothesised that employment and household wealth would be inversely associated with the experience of violence at both levels of analysis and that this association would persist after controlling for individual and household characteristics.

## Methods

The SADHS was a national household survey conducted during 1998. Sampling was conducted in two stages. First, the country was stratified into urban and non-urban areas by province. Then enumerator areas (EA, the unit of census administration) were selected within each stratum. To ensure that robust estimates for each population group and each province were achieved areas with a high density of Asians and smaller provinces were over-sampled. (The *Apartheid* regime in South Africa sought to categorise all South Africans into one of four racial groups; racial group stratification has been retained in national health surveillance in South Africa to reflect a social complexity not fully captured by standard socioeconomic measures as well as to monitor progress toward reduction of health disparities.) The second stage of sampling was the selection of 20 visiting points in each non-urban EA and ten visiting points in each urban EA. Sampling without replacement was used and up to three attempts were made to contact the selected individuals. Trained interviewers administered the questionnaires in one of the 11 official South African languages. Ethical approval to conduct the SADHS was provided by the Ethics Committee of the South Africa Medical Research Council and all participants provided verbal informed consent prior to being interviewed.

### Measures of violence

The household survey of the SADHS collected information on the experience of an intentional injury that resulted in treatment by a doctor or nurse in the past 30 days. The household respondent reported this information for every member of the residence. Categories of intentional injuries were: assault in the home, political violence, other assault outside of home, and self-inflicted violence. For analysis, political violence (n = 1) was combined with violence outside the home and reports of self-inflicted violence were excluded from the dataset (n = 23). Violence outside the home was combined with violence inside the home (n = 88), producing a binary variable.

The number of intentional violent deaths occurring within the household in the past year ranged from zero to three per household. Violent deaths caused by self-inflicted violence were included due to the nature of the survey question, and could not be excluded from analysis. Households that experienced one or more violent death (range:1,3) were grouped together to create a binary variable (n = 103). Information on the type of violence leading to death and individual characteristics of the member who died was not collected.

### Socioeconomic position

Measures of socioeconomic position included education, employment and wealth of the household. For the last we used an asset index based on household characteristics and possessions. An asset index is frequently used as a measure of absolute deprivation in Demographic and Health Surveys in the place of measures of individual or household income, which may not adequately represent wealth in many settings [13]. The index used for this analysis is based on a preliminary factor analysis of 55 wealth-related variables. When entered into a factor analysis 14 variables received loadings greater than |0.50|: electricity; ownership of television, refrigerator, car, telephone, washing machine; use of electricity or wood for cooking; use of electricity or wood for heating; presence of piped drinking water in dwelling; has flush toilet, earth floors, mud walls, plastered walls; and family members never go hungry. These items were combined into a single aggregate measure; households were ranked using the index, and then divided into quintiles from poorest (“1”) to wealthiest (“5”).

Employment status was collected on participating household members aged ten years or older (n = 39,008). Employment was defined as working for payment in the previous seven days. The total years of formal schooling was collected on all members of the household (n = 52,906).

### Statistical analysis

Data were analysed using Stata Version 8.0 (College Station, Texas, USA). All analyses include survey-based weights which accounted for the complex survey design. Unadjusted analyses were conducted to identify potential determinants of experience of violence at individual and household levels. At the individual level, a comparison of means and proportions for the receipt of treatment for an intentional injury in the past 30 days was carried out in relation to the following variables: age, education, sex, race (Black/African, Coloured, Indian/Asian, White), employment, and involvement in a medical insurance scheme. At the household level, odds ratios (OR) and 95% confidence intervals (CI) were estimated for violent death in the past year using the following determinants: the sex, age, race, employment status and education of the head of household; and asset index quintile of household.

Separate multiple logistic regression models were developed to examine individual experience of violence and household experience of violent death as categorical dependent variables. Automated model building procedures (forward, backward or stepwise regression) are not supported by the survey analysis command in Stata 8.0 and are therefore not used in any of the analyses. A controlled model building procedure was utilised to ensure that key confounding variables were included in the analysis and hypotheses around mediation could be tested.

Owing to the strength of the association between employment and violence at the bivariate level, employment was used as the key independent variable in both multivariate models. The models originated with this bivariate association and known demographic confounding variables were added to the model individually. At the household level data on employment, education and demographics was based on that of the household head. The second step was to assess the association between the original measure of socioeconomic position and violence once other measures of socioeconomic position were added to the model (education of the individual or household head and the asset index).

Standard model diagnostic procedures adapted for multivariate models [14], [15] cannot be applied to survey data in Stata 8.0, therefore the adequacy of model fit and other diagnostic procedures such as assessing the normality and variance of residuals could not be assessed. Due to the small number of cases of violence, analysis of influence through the removal of specific cases or observations [16] would not have been useful, as the removal of any would have altered results drastically.

## Results

A total of 12,247 households were successfully interviewed, with 13,826 adults responding to the adult questionnaire (administered only in every other household). Response rates varied between 92% and 97%.

Table 1 describes the 52,906 individuals residing in the 12,247 households participating in the SADHS. Almost half of these individuals were under the age of 20 years and 53% were female. Over one-quarter of individuals older than age of ten years had worked in the past seven days and nearly 80% were Black/African. Head of households had a mean age of 49 years, over half had a primary education or less and 46% had worked in the past seven days. Eighty-eight people (0.2%) had experienced a violent injury in the past 30 days and received medical treatment, and 0.9% of households had experienced a violent death in the past year (n = 103). When including both injuries and deaths, 1.5% of households had experienced violence (in the last month or year respectively).

**Table 1:** Description of individual and household study sample

Individual-level variables (n = 52 906 unless otherwise noted)	% (95% CI)	Household-level variables (n = 12 247 unless otherwise noted)	% (95% CI)
Total interpersonal violence	0.16 (0.12, 0.20)	Experienced a death due to violence in the past year	0.89 (0.71, 1.11)
Violence outside the home	0.11 (0.09, 0.15)	Experienced a violent death or injury in the past year/month	1.53 (1.29, 1.81)
Violence inside the home	0.04 (0.03, 0.07)		
Worked in the past 7 days (n = 39 008)	27.45 (26.37, 28.56)	Head of household worked in the past 7 days (n = 12 030)	45.98 (44.26, 47.71)
Highest level of schooling primary or less	60.85 (59.79, 61.91)	Head of household primary level education or less	51.15 (49.63, 52.66)
Male	46.74 (46.25, 47.23)	Female headed household	41.86 (40.56, 43.18)
Race (n = 47 091)		Race of household head (n = 10 320)	
Black/African	79.66 (77.64, 81.55)	Black/African	77.05 (74.96, 79.01)
Coloured	9.97 (8.62, 11.49)	Coloured	9.89 (8.56, 11.28)
White	7.24 (6.11, 8.32)	White	9.84 (8.39, 11.51)
Asian/Indian	3.13 (2.38, 4.10)	Asian/Indian	3.23 (2.47, 4.21)
Age group (years)		Mean age of head of household (years)	48.51 (48.03, 48.98)
0–19	47.72 (47.04, 48.40)	Household asset index (n = 12 017)	
20–29	15.23 (14.79, 15.67)	1 (Poorest quintile)	14.84 (13.56, 16.22)
30–39	12.13 (11.74, 12.53)	2 (2^nd^ poorest quintile)	21.52 (19.96, 23.16)
40–49	8.71 (8.36, 9.13)	3 (Middle quintile)	19.29 (17.86, 20.79)
50–59	7.50 (7.17, 7.84)	4 (2^nd^ richest quintile)	20.74 (19.22, 22.34)
60+	8.72 (8.33, 9.13)	5 (Richest quintile)	23.62 (21.76, 25.58)
Medical insurance (n = 13 780)	17.05 (16.27, 17.87)		

CI : Confidence interval

Table 2 displays the individual-level risk factors associated with receiving treatment for a violent injury in the 30 days prior to the survey. Individuals who did so were more likely to be male and older. They also had an overall average of one year more of education and were more likely to be employed. Coloured respondents were somewhat overrepresented among those who experienced violent injury.

**Table 2:** Individual level risk factors for receipt of treatment by a doctor or nurse for violence-related injury in the past 30 days

Risk factor	Sample size of dataset used	No violence	Any violence	p-value*
		**Mean**		
Age (years)	52 906	26.30	30.04	0.02
Education (years)	52 906	5.71	6.82	0.04
		**%**		
Male	24 721	46.71	65.30	<0.01
Race				0.32
Black/African	36 976	79.66	77.57	0.67
Coloured	5 793	9.96	16.44	0.07
Indian/Asian	2 894	3.14	0	0.34
White	1 425	7.25	5.99	0.70
Employed**	9 971	27.42	41.32	0.02
Involvement in a medical insurance scheme***	2 039	17.06	10.49	0.28

* P-value for Pearson's chi-square test of homogeneity

** Sub-population of all persons aged 10 years and older

*** Sub-population of persons aged 15 and older in every other household selected

Table 3 presents the associations between household-level variables and death due to violence in the household in the past year. Female-headed households were 80% more likely to have experienced a death due to violence than households headed by a male. When compared to Black/African households, both White/Asian and Coloured households had a lower experience of violent death. Surprisingly, the asset index did not display a trend of decreasing risk of violent death with increasing wealth; in fact, the fourth quintile showed a sharp increase in the point estimate. In contrast, increasing education and employment of the head of household reduced the odds of death in the household in the past year.

**Table 3:** Bivariate analyses of household characteristics and the occurrence of a violent death in the previous year

Dependent Variable	Sample size of dataset used	Violent death (n = 103) Odds ratio (95% CI)	P-value*
Sex of head of household	12 247		
Male headed household		1.0	
Female headed household		1.81 (1.14, 2.87)	0.01
Age of head	12 247	1.02 (1.01, 1.03)	0.01
Race of head of household	10 320		
Black/African		1.0	
Coloured		0.40 (0.16, 0.97)	0.04
White/Asian**		0.07 (0.01, 0.48)	0.01
Asset index quintile	12 017		
Poorest quintile		1.0	
2^nd ^poorest quintile		0.99 (0.53, 1.86)	0.99
Middle quintile		0.57 (0.27, 1.19)	0.14
2^nd^ richest quintile		1.72 (0.93, 3.18)	0.09
Richest quintile		0.22 (0.07, 0.70)	0.01
Employment of head	12 030		
No work for payment in the past 7 days		1.0	
Worked for payment in the past 7 days		0.36 (0.20, 0.63)	<0.001
Years of education of head	12 247	0.95 (0.91, 0.99)	0.01

* P-value for Pearson's chi-square test of homogeneity

** Owing to the small sample size White and Indian/Asian population groups were combined for analysis

The adjusted effects of socioeconomic position (employment, education and household wealth) on the individual experience of violence are displayed in Table 4. The positive effect of employment was enhanced with the addition of demographic characteristics to the model (Model 1). The effect persisted when other measures of socioeconomic position were added (education and household wealth) although it was no longer statistically significant (OR: 1.83; 95% CI: 0.99–3.38; Model 2). Likewise, the elevated risk associated with the Coloured population group remained but was not statistically significant (OR: 1.94; 95% CI: 0.95–3.96). Living in a household in the wealthiest quintile compared to living in a household in the poorest quintile was significantly protective against experience of violence.

**Table 4:** Multivariate analyses of effect of socioeconomic position and receipt of treatment for an intentional injury in the past month

Model variables	Crude odds ratio	Model 1	Model 2
	(95% CI)	OR (95% CI)	OR (95% CI)
	n = 38 992	n = 34 262	n = 33 367
Employed	1.86 (1.12, 3.11)	2.09 (1.18, 3.70)	1.83 (0.99, 3.38)
Age (years)		0.987 (0.976, 0.999)	0.99 (0.98, 1.00)
Male		2.25 (1.27, 3.98)	2.49 (1.37, 4.54)
Race			
Black/African		1.0	1.0
Coloured		1.34 (0.66, 2.71)	1.94 (0.95, 3.96)
White/Asian*		0.33 (0.10, 1.09)	0.47 (0.13, 1.68)
Education (years)			1.08 (0.99, 1.18)
Asset index quintile			
Poorest quintile			1.0
2^nd^ poorest quintile			0.62 (0.24, 1.61)
Middle quintile			0.66 (0.29, 1.50)
2^nd^ richest quintile			0.56 (0.19, 1.62)
Richest quintile			0.32 (0.12, 0.89)

OR: Odds ratio; CI: Confidence interval

* Owing to the small sample size White and Indian/Asian population groups were combined for analysis

Table 5 illustrates the effect of socioeconomic position of the head of household on the occurrence of a violent death in the household in the past year. In crude analysis, employment of the household head reduced the odds of a violent death by 64%; after adjustment for demographic characteristics of the household head, the protective odds were no longer significant (Model 1). The only statistically significant variable in this model was the preventive effect of membership within a White/Asian household when compared to a Black/African household (OR: 0.10; 95% CI: 0.01–0.78). When other measures of socioeconomic position were added to the model (education of the household head and household wealth), the point estimate for employment of the household head changed only slightly although the confidence interval was considerably wider (Model 2). There was no consistent association with household wealth.

**Table 5:** Multivariate analysis of the effect of household socioeconomic position on the experience of a violent death within the household in the previous year

Model variables	Crude odds ratio	Model 1	Model 2
	(95% CI)	OR (95% CI)	OR (95% CI)
	n = 12 030	n = 10 129	n = 9 833
Employed head	0.36 (0.20, 0.63)	0.50 (0.25, 1.00)	0.51 (0.25, 1.04)
Age of head (years)		1.01 (0.996, 1.03)	1.01 (0.99, 1.03)
Male head		0.66 (0.34, 1.16)	0.63 (0.36, 1.13)
Race of head of household			
Black/African		1.0	1.0
Coloured		0.53 (0.21, 1.33)	0.54 (0.21, 1.40)
White/Asian*		0.10 (0.01, 0.78)	0.16 (0.01, 1.85)
Education of head (years)			(0.95, 1.08)
Asset index quintile			
Poorest quintile			1.0
2^nd^ poorest quintile			1.00 (0.50, 2.00)
Middle quintile			0.57 (0.24, 1.35)
2^nd^ richest quintile			2.03 (0.98, 4.21)
Richest quintile			0.57 (0.09, 3.55)

OR: Odds ratio; CI: Confidence interval

* Owing to the small sample size White and Indian/Asian population groups were combined for analysis

## Discussion

Levels of violence are high in South Africa. In this study we have found that in 1998, 2 in 1000 South Africans of across ages experienced a violent injury requiring medical treatment in the previous month while 9 in 1000 households had a violent death in the previous year. Considering the individual level data provided in the SADHS only reflect those events which resulted in the receipt of medical care, actual rates of violence during this time period are likely to be much higher.

With respect to socioeconomic position, we found that employment and education were risk factors for violence at the individual level, whereas being in the wealthiest quintile was protective against violence. In contrast, at the household level, all three measures were protective against the experience of a violent death. While these associations did not remain statistically significant in the multivariate analysis (with the exception of the wealthiest index at the individual level), the trend remained and the lack of significance may be explained by the reduced number of injuries/deaths in the dataset.

The inverse relationship found between measures of socioeconomic position and violent death at the household level was expected. The discrepancy between the two levels of analysis may suggest that the relationship between socioeconomic position and violent outcomes is dependent on the severity of the outcome (fatal vs. nonfatal). However, as the employment status of the individual who died was unknown, the relationship between individual experience of fatal injury and personal employment could not be tested. This, as well as other differences between the two outcome measures such as the difference in time period between the measures and the inclusion of self-inflicted violence at the household level, makes the influence of injury severity on these associations difficult to determine.

The findings with regard to the association between race and the experience of violence also differed between the individual and household levels. The increased risk of receipt of treatment for violence that was found for the Coloured population may be explained by the higher rates of violence in the Western and Northern Cape Provinces (SADHS data, not shown). Over half the population in each of these provinces self-identifies as Coloured [17], and these provinces have previously been found to be the most violent [18], [19]. This finding was not replicated for the household analysis where Black/African households were more likely to have experienced a violent death.

These data suggest that being employed was significantly associated with risk of experiencing violence, particularly with respect to violence that occurred outside the home. This finding may be explained in part by the risk associated with working due to commuting, working in high risk jobs, or having more money (or material possessions) and thus becoming a target for violent crime. Employment was based on work for payment in the previous seven days, which may not have been an adequate proxy for employment status. However, it is likely that any such misclassification would have been non-differential with respect to experience of violence, thereby diluting the effects and would thus not explain the differential effects of employment that were found here.

Similar to employment, there was a positive correlation between increasing education and the receipt of treatment for an intentional injury. When demographic characteristics and other measures of socioeconomic position were added to the model (Table 4 Model 2), education was no longer a significant risk factor for violence. This could be an indication that employment mediated the relationship between education and violence.

This analysis is subject to a number of limitations. We are unable to definitively establish the temporality of the associations between injury and socioeconomic position, as it is possible that injury may have prevented employment, or less likely, loss of material wealth. However as socioeconomic position is relatively fixed, and we used measures of violence in the past 12 months, this is unlikely to account for the associations observed here. In addition, with only 88 injuries and 103 deaths, we had limited statistical power to detect small associations involving violence.

As mentioned, individual-level results display the observed associations between socioeconomic position and the receipt of treatment for an intentional injury rather than the actual experience of an injury. Those who receive an injury but do not get treatment may be different than those that do in terms of wealth, access to care or tolerance for pain/violence. It is important to note, however, that only 17% of the study sample was involved in a medical aid scheme and this was not a significant correlate with the outcome at the bivariate level. As collected by the SADHS, data on the individual experience of violence may have biased our results toward an underestimation of violent occurrences in the less wealthy population (and dilute the inverse relationship between wealth and the experience of violence that we hypothesised). However, all measures of socioeconomic position did not have the same (positive) direction of association with violence as could be expected if the well off were more likely to receive treatment for a violent injury.

This research provided an overview of the national epidemiology of violence in South Africa. The 1998 SADHS is the first of it's kind in South Africa and is one of the few population-based datasets in sub-Saharan Africa that can be used to examine risk factors for violence. Importantly, this dataset includes non-fatal violence, an outcome on which there are few data from developing country settings, where violence research typically focuses primarily on violent death [20].

Given that employment and higher level of education were identified as risk factors for the experience of violence at the individual level in this analysis, potential mediating variables need to be identified and further explored so this relationship is better understood. Such mediating variables may include unsafe public transportation areas or the increased use of alcohol that could lead to an increase in risk for violence if one is employed. Given the prevalence of violence at taxi cab ranks in South Africa [21], as well as in the informal sector [22], the role that modes of transportation and different types of employment play in violent injuries also deserves exploration.

In summary, this analysis provides insight into the intricacies between socioeconomic position, the ability and desire to seek medical treatment for an injury and the experience of a violent injury. While more data is needed to draw out these linkages, this analysis has begun to identify the disparities across the socioeconomic structure with respect to violent outcomes.
